# Characterization of the complete chloroplast genome of *Salix koreensis* Anderss (Salicaceae) 1868

**DOI:** 10.1080/23802359.2022.2087561

**Published:** 2022-06-23

**Authors:** Jintai Wu, Xiaoping Li

**Affiliations:** a Collaborative Innovation Center of Southern Modern Forestry, Nanjing Forestry University, Nanjing, China; b College of Forestry, Nanjing Forestry University, Nanjing, China; c Jiangsu Key Laboratory for Poplar Germplasm Innovation and Variety Improvement, Nanjing Forestry University, Nanjing, China

**Keywords:** *Salix koreensis* Anderss, chloroplast genome, phylogenetic analysis

## Abstract

*Salix koreensis* Anderss 1868 is a grey-barked, arboreal willow with high economic value. This study reports the *S. koreensis* chloroplast (cp) genome. A phylogenetic tree has also been constructed to illustrate the relationship between *S. koreensis* and 25 other species within the Salicaceae family. The total length of the chloroplast genome is 155,865 bp, which can be divided into four parts: one large single-copy (LSC) length of 84,477 bp, one small single-copy (SSC) length of 16,316 bp, and two inverted repeat (IRA and IRB) lengths of 27,446 bp. The cp genome of *S. koreensis* is composed of 133 genes, including 86 protein-coding genes, 38 tRNA genes, and 8 rRNA genes. According to the phylogenetic tree, *S. koreensis* and *S. chienii* are closely related.

*Salix koreensis* Anderss is a deciduous, broad-leaved tree species in the family Salicaceae and the genus *Salix*. This species of willow grows on riversides and hillsides at an altitude of 50–700 meters. *S. koreensis* is naturally distributed in some northeastern provinces of China such as Heilongjiang, Jilin, and Liaoning. It can also be found in some areas of Korea, Japan, and Russia (Wang and Fang 1984). The twig bark of S. *koreensis* is known to be effective for pain relief in Korean, making this plant useful for medicinal applications (Yun et al. 2016). Additionally, *S. koreensis* is advantageous in forest fire prevention because its bark has low flammability (Shan et al. 2012). However, the current research available on *S. koreensis* is still scarce, particularly in terms of its molecular biology. The current study was the first to characterize the chloroplast genome of *S. koreensis*, providing genomic information for future studies. A phylogenetic tree was also constructed to demonstrate the evolutionary relationship between *S. koreensis* and 25 other plants in the Salix genus.

Fresh and healthy leaves of *S. koreensis* were collected from Maoer Mountain (44°29′29″ N, 127°17′41″ E), which is located in Harbin City in Heilongjiang Province, China. The voucher specimens (voucher number: MRSCXL2017_64) were deposited in the herbarium of Nanjing Forestry University (https://www.njfu.edu.cn, Xiaoping Li, xpli@njfu.edu.cn). The genomic DNA was extracted from the fresh leaves of *S. koreensis* using the Cetyl Trimethyl Ammonium Bromide (CTAB) method (Doyle and Doyle 1987). The derived DNA was used to construct a genome library using 2 × 150 bp paired-end (PE) sequencing on the Illumina NovaSeq 6000 Platform (Illumina, San Diego, CA). Approximately, 13.34 G raw reads were obtained from sequencing, and Fastp (Chen et al. 2018) was then used for trimming. The remainder of the reads (approximately 11.65 G) were clean reads. The circular chloroplast genome sequence of *S. koreensis* was obtained using NOVOPlasty4.1 (Dierckxsens et al. 2017) to assemble clean reads. Due to the high conservation in the chloroplast genome of the genus Salix, Basic Local Alignment Search Tool(BLAST) was used to search *S. chienii* (GenBank accession number: MW969692) as a reference, and the sequence of *S. koreensis* was annotated using Plastid Genome Annotator (PGA) (Qu et al. 2019). The final annotation and manual correction were conducted using Geneious software (Kearse et al. 2012). Finally, the complete cp genome sequence of *S. koreensis* and its annotation were submitted to GenBank (accession number: OK500208).

The total length of the *S. koreensis* chloroplast genome is 155,685 bp with 36.6% GC content. The cp genome exhibits a typical quadripartite circular structure with four distinct regions. These consist of a pair of inverted repeat regions (IRB and IRA), one small single-copy (SSC) region, and one large single-copy (LSC) region with lengths of 84,477, 16,316, and 27,446 bp, respectively. GC content accounts for 34.4, 31.0, and 41.7% of these sequences, respectively. The cp genome of *S. koreensis* contains 133 annotated genes, including 86 protein-coding genes, 38 transfer RNA (tRNA) genes, and 8 ribosomal RNA (rRNA) genes.

To investigate the phylogenetic relationship between *S. koreensis* and other *Salix* species, a phylogenetic tree was created using a maximum-likelihood algorithm. The complete cp genome sequence of *S. koreensis* and 25 other *Salix* species were aligned using MAFFT v7 software (Katoh and Standley 2013). *Hevea brasiliensis* (Tangphatsornruang et al. 2011) was selected as the outgroup. IQ-TREE software (Nguyen et al. 2015) was utilized to produce the phylogenetic tree using a best-fit model of K3Pu + F+R7 and 1,000 bootstrap replicates. Based on the tree (Figure 1), *S. koreensis* and *S. chienii* are closely related, with strong support values (BP = 100%). *S. chienii* is a willow tree with high medical value. It can be found along streams at an altitude of 500–600 meters in several provinces of China including Anhui, Zhejiang, Hunan, Hubei, Jiangsu and Jiangxi (Wang and Fang 1984). The results of this study could provide the basis for future plant classification of the genus *Salix*, gene annotation, and molecular identification.

**Figure 1.**
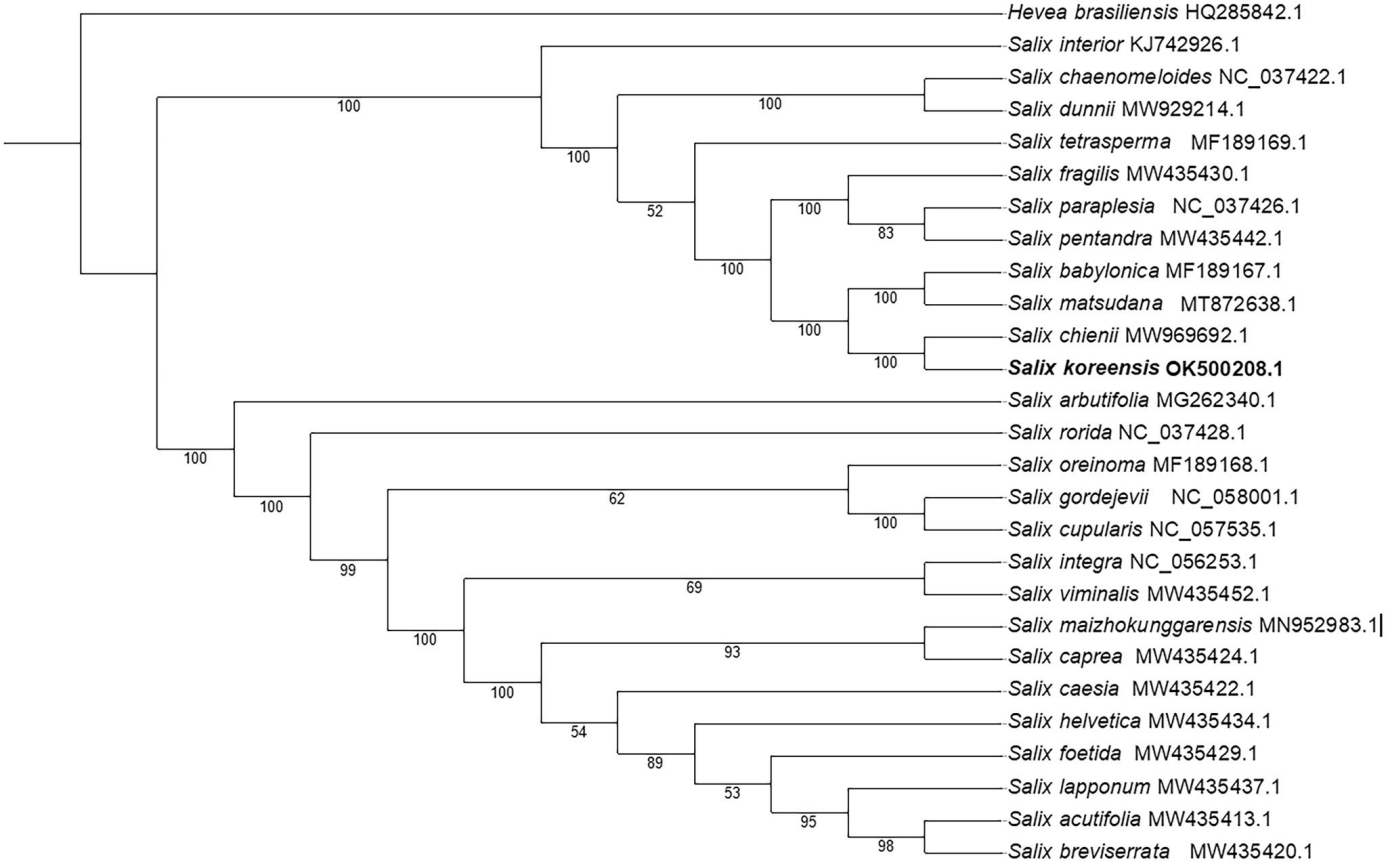
A phylogenetic tree based on the chloroplast genome sequences of 26 Salicaceae species with 1,000 bootstrap replicates. The bootstrap support values are displayed under each branch.

## Data Availability

The genome sequence data that support the findings of this study are openly available in GenBank of NCBI (https://www.ncbi.nlm.nih.gov/) under the accession number of *S. koreensis* is No.OK500208. The associated Bioproject, Biosample, and SRA numbers are PRJNA771624, SAMN22322916, and SRR16531625 respectively.
